# Safety and efficacy of plasma exchange therapy for Kawasaki disease in children in intensive care unit: case series

**DOI:** 10.1186/s40981-018-0156-3

**Published:** 2018-03-06

**Authors:** Satoko Noguchi, Junichi Saito, Tomoyuki Kudo, Eiji Hashiba, Kazuyoshi Hirota

**Affiliations:** 1 Department of Anesthesiology, Hirosaki University Graduate School of Medicine, 5 Zaifucho, Hirosaki, 036-8562 Japan; 2 Department of Intensive Care Unit, Hirosaki University Medicine Hospital, Hirosaki, Japan

**Keywords:** Kawasaki disease, Pediatric intensive care, Plasma exchange therapy

## Abstract

**Background:**

We have reviewed four cases of Kawasaki disease treated with plasma exchange with 5% albumin in electrolyte-balanced solution, according to the recommended guidelines for Kawasaki disease in the intensive care unit, as their responses to intravenous immunoglobulin therapy were poor.

**Case presentation:**

The four cases were aged between 5 months and 3 years and weighted between 6.4 and 15.6 kg. The plasma levels of C-reactive protein were significantly decreased after plasma exchange (*p* < 0.05). The dilatations of the coronary artery were found in two cases, but both of them were ameliorated until 1 month after the onset and the other cases recovered without any complications. However, we recognized that one case showed marked decreases in coagulation factors, especially in fibrinogen after each plasma exchange even with a transfusion of fresh frozen plasma.

**Conclusions:**

Plasma exchange with 5% albumin was effective for refractory Kawasaki disease. However, as there was a possibility of coagulation disorder, attention should be given to changes in coagulation factors like fibrinogen, especially in small patients who need frequent plasma exchange.

## Background

Kawasaki disease (KD) is a systemic vasculitis of unknown cause, which often occurs in young children. Coronary artery disease and myocarditis are fatal complications of KD, and early application of high-dose intravenous immunoglobulin therapy (IVIG) is effective for the majority of the KD. However, some forms of KD are not responsive to the IVIG therapy and alternative therapies such as immunosuppressive therapies and plasma exchange (PE) therapy are applied. PE for KD has been approved as an official alternative therapy by the national health insurance system in Japan since 2012, and the number of application of PE has increased. However, PE therapy itself has been known to be problematic because of hemodynamic instability, coagulation disorders, and potential infections especially in small children. Sedation in children during the PE therapy has been also controversial. Therefore, we have reviewed four cases of KD, which were refractory to IVIG therapy and treated with PE in the intensive care unit, in terms of safety and efficacy of PE.

## Case presentation

Following approval by the Medical Ethics Committee of Hirosaki University Graduate School of Medicine (approval number 2017-1025), medical records of patients receiving PE for KD in our intensive care unit (ICU) between January 2014 and April 2017 were retrospectively reviewed. PE was indicated for the patients who were considered to be refractory to IVIG therapy by pediatricians because initial and additional IVIG treatments were ineffective or abnormalities of the coronary arteries had already developed after the initial IVIG.

PE was generally conducted as a vein-to-vein procedure, and a 7–8 Fr double-lumen dialysis catheter was inserted into a femoral vein and the plasma was separated using a plasma-separating membrane (Plasmaflo® OP 0.2~0.5 W, Asahi Kasei Medical, Tokyo). Five percent albumin in electrolyte-balanced solution was used as the replacement fluid according to the guidelines for KD [1]. The amount of exchanged plasma was approximately 1.0–1.3-fold of circulating blood plasma volume. Nafamostat mesylate (0.5~ 1.0 mg/kg/h) was used as an anticoagulant, and the activated clotting time was adjusted to remain between 150 and 200 s.

PE was performed for 3 days consecutively, but if there was no decrease in temperature or improvement in the inflammatory state, the duration of PE was extended to a maximum of 5 or 6 days. The following data were collected from each patient’s medical record: age, sex, body weight, height, comorbidity like congenital disease, asthma and epilepsy, total dose of IVIG before PE, the other treatments before PE, the day when PE was introduced after the onset of the KD, development of coronary artery lesions, left ventricular ejection fraction (%) estimated by echocardiography, the data related to PE such as duration of PE, data of inflammation makers, method of PE, type of sedation, respiratory and circulatory management, blood transfusions, length of hospital stay, morbidity, and mortality.

In order to evaluate the safety and efficacy of PE for children with KD, we compared the changes in inflammatory parameters and coagulation factors. Statistical analysis was performed using GraphPad Prism 5 (GraphPad Software Inc., San Diego, CA, USA). Paired *t* test was used to compare the data before and after PE, taking *p* < 0.05 as significant.

The review included four children aged between 5 months and 3 years and weighed between 6.4 and 15.6 kg (Table 1). The initial doses of IVIG were given at 4 or 5 days after the onset of the KD, and 3 or 4 g/kg of IVIG was given prior to PE. PE was introduced 8 to 10 days after the onset, and the duration of PE treatment was between 3 and 5 days. Continuous intravenous administration of dexmedetomidine (0.2–0.7 μg/kg/h) without a loading dose and bolus administration of midazolam (0.5–1.0 mg) were used for cannulation of vascular access, together with infiltration of local anesthesia. This combination of sedation was used for PE therapy if needed, but no patient required ventilator management or catecholamine administration during PE. We introduced PE into these pediatric patients in ICU, including the cannulation of a vascular access and made them stay in ICU just one night. Following their second PE, all patients were discharged to general pediatric ward because the patients could spend their time with their parents and unnecessary sedatives could be spared. Patients then visited ICU for further treatment with PE.

**Table 1:** Patient demographics and management of PE

No.	1	2	3	4
Age	7 months	2 years 8 months	3 years	5 months
Sex	Male	Female	Male	Male
Height (cm)	72	92	89	64
Weight (kg)	8.8	15.6	14.7	6.4
Comorbidity	None	None	None	None
Total dose of IVIG (g/kg)	3	4	4	4
The day on which PE started	Day 10	Day 9	Day 9	Day 8
EF before PE (%)	75	63	70	69
Coronary artery lesions				
Before PE	None	Dilation	None	None
After PE	None	Dilation	Dilation	None
Sedation during PE				
DEX (μg/kg/h)	0.4–0.7	0.4–0.6	0.4–0.7	0.2–0.7
Respiratory management	0.5 L/min	RA	RA	6 L/min
PE duration (days)	3	3	5	5
Transfusion				
RBC	60 mL	None	240 mL	90 mL
FFP	None	None	None	320 mL
Length of hospital stay (days)	6	15	14	16
Hemorrhagic complications	None	None	None	None
Other complications	None	None	None	None

*IVIG* intravenous immunoglobulin therapy, *PE* plasma exchange, *EF* light ventricular ejection fraction, *DEX* dexmedetomidine, *RA* room air, *RBC* red blood cell concentration, *FFP* fresh frozen plasma

**Fig. 1:**
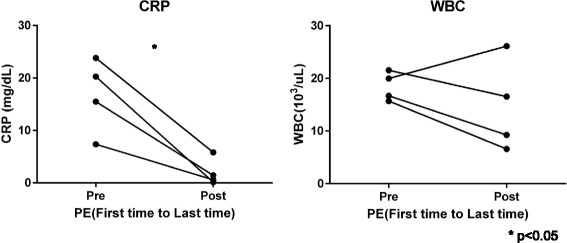
Inflammatory response change before and after PE. C-reactive protein was decreased significantly (*p* < 0.05)

After a series of PE, plasma levels of C-reactive protein were significantly decreased (Fig. 1). Coronary artery dilatation developed in two patients, one of which was found before the initiation of PE (no. 2) and the other was found during PE (no. 3). Fortunately, both of them were ameliorated and there was no complication associated with PE.

**Fig. 2:**
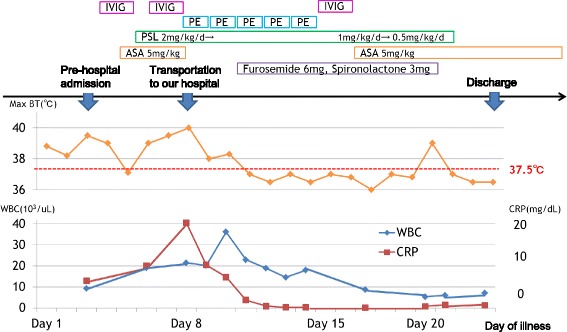
Progress course of case no.4. After PE, both inflammatory data and body temperature decreased promptly

**Fig. 3:**
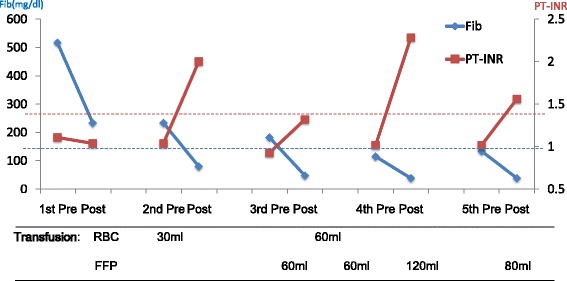
Coagulation changes for each PE of case no.4. The more PE was performed, the more the coagulation ability becomes abnormal even if the patient was transfused

The clinical course of no. 4 patient who was the youngest and smallest of the four patients was shown in Fig. 2. High body temperature decreased and inflammation improved following PE. However, there was a marked increase in PT and a decrease in fibrinogen, after each PE, even if the patient was transfused (Fig. 3).

## Conclusions

We report four cases of KD that required PE therapies performed with 5% albumin instead of fresh frozen plasma (FFP), according to the guidelines for KD [1]. Although there were no severe complications, one case showed a marked disturbance of coagulation factors, such as fibrinogen even with FFP transfusion after PE.

PE is an alternative therapy for KD refractory to IVIG therapy to prevent coronary artery lesions [2]. Suppressing the inflammatory response at an early stage was considered to prevent coronary complications. Therefore, PE is recommended to be introduced at a relatively early stage, soon after fractional increases in inflammatory markers are found following the first or second IVIG therapy. It has been also reported that myocarditis coincident with KD was successfully treated with PE [3]. Since PE may cause hemodynamic instability, especially when cardiac function is impaired by myocarditis, the slow plasma exchange therapy combined with continuous hemodiafiltration has also been reported as an effective safe method [4]. In our cases, the inflammatory responses including C-reactive protein significantly decreased after PE and two coronary artery lesions were ameliorated in line with previous reports [5, 6].

According to the guidelines for treatment of KD [1], albumin solution is recommended as a replacement fluid. In our hospital, 5% albumin in electrolyte-balanced solution was prepared with mixture of 20% albumin, lactic Ringer’s solution, NaCl, and CaCl_2_ to adjust electrolyte concentration. However, as seen in case no. 4, PE with albumin disturbed coagulation ability (Fig. 3). Although Taguchi et al. suggested a probability of coagulation disturbance after PE with albumin [7], there was no detailed data before and after each PE in their report. We did not experience hemorrhagic complications in our cases, but it is important to observe the coagulation state around PE therapy with albumin. As Witt et al. have warned, it should be considered that FFP transfusion or PE with FFP is occasionally performed depending on the coagulation state before PE [8]. Selective PE using a special plasma separator, which can remove albumin with antibodies and cytokines, but retaining coagulation factors like fibrinogen, is an alternative method. However, a selective PE membrane is only available for adult patients in Japan [9]. Fujimaru et al. have suggested that removal of inflammatory cytokines was the most important role of PE for refractory Kawasaki disease [10]. Therefore, the development of a selective PE membrane for small children is necessary.

In terms of secure performance of PE in children, immobilization during cannulation of a vascular access and PE therapy is necessary. Koizumi et al. suggested a sufficient sedation for PE even with a mechanical ventilator and the use of catecholamine [4]. In our institution, the cannulation and PE therapy were performed using continuous administration of dexmedetomidine and bolus administration of midazolam without mechanical ventilation. Buck et al. reviewed studies evaluating the safety and efficacy of dexmedetomidine in infants and children and concluded that dexmedetomidine was an additional choice for the sedation of children not only receiving mechanical ventilation, but also requiring procedural sedation in ICU [11]. We carefully monitored the patients’ respiratory and circulatory vital signs during PE and made the patients stay just one night in ICU after the first PE to avoid unnecessary sedation, and to be with their parents for comfort. The patients were transferred to pediatric wards, and visited ICU each day, as required, for PE therapy, although there was an economical problem in that an ICU administration fee could not be claimed for the patient according to the Japanese national health insurance system.

PE with 5% albumin was effective for refractory KD, with or without coronary complications, as previously reported. However, careful observation of coagulation factors, such as fibrinogen, is advised to due to the risk of coagulation disorder, especially in children who require repeated applications of PE.

## Abbreviations

FFP: Fresh frozen plasma
IVIG: Intravenous immunoglobulin therapy
KD: Kawasaki disease
PE: Plasma exchange
